# Screening of primary aldosteronism and pheochromocytoma among patients with hypertension: an Italian nationwide survey

**DOI:** 10.1007/s40618-025-02532-5

**Published:** 2025-01-18

**Authors:** Silvia Monticone, Jessica Goi, Jacopo Burrello, Guido Di Dalmazi, Arrigo F. G. Cicero, Costantino Mancusi, Elena Coletti Moia, Guido Iaccarino, Franco Veglio, Claudio Borghi, Maria L. Muiesan, Claudio Ferri, Paolo Mulatero, Italian Society of Arterial Hypertension (SIIA)

**Affiliations:** 1 Division of Internal Medicine 4 and Hypertension Unit, Department of Medical Sciences, University of Torino, Torino, Italy; 2 Division of Endocrinology and Diabetes Prevention and Care, IRCCS Azienda Ospedaliero- Universitaria di Bologna, Bologna, Italy; 3 Department of Medical and Surgical Sciences (DIMEC), Alma Mater Studiorum University of Bologna, Bologna, Italy; 4 Hypertension and Cardiovascular Risk Research Unit, Medical and Surgery Sciences Department, Alma Mater Studiorum University of Bologna, Bologna, Italy; 5 Department of Advanced Biomedical Sciences, Federico II University of Naples, Via S. Pansini 5, 18, Napoli, 80131 Italy; 6 ARCA (Associazioni Regionali Cardiologi Ambulatoriali) Piemonte, Turin, Italy; 7 Department of Clinical and Experimental Sciences, University of Brescia, Brescia, Italy; 8 Department of Life, Health and Environmental Sciences, University of L’Aquila, L’Aquila AQ, Italy

**Keywords:** Primary aldosteronism, Pheochromocytoma, Secondary hypertension, Aldosterone to renin ratio

## Abstract

**Purpose:**

The delayed or missed diagnosis of secondary hypertension contributes to the poor blood pressure control worldwide. This study aimed to assess the diagnostic approach to primary aldosteronism (PA) and pheochromocytoma (PHEO) among Italian centers associated to European and Italian Societies of Hypertension.

**Methods:**

Between July and December 2023, a 10-items questionnaire was administered to experts from 82 centers of 14 Italian regions and to cardiologists from the ARCA (Associazioni Regionali Cardiologi Ambulatoriali) Piemonte. Results were stratified for geographical area, specialty, and center category (excellence vs. non-excellence centers).

**Results:**

Each center diagnosed an average of 2 cases of PA and 0.2 cases of PHEO annually, with higher figures in excellence centers. PA screening is performed mainly in patients with resistant hypertension (73.2%) or hypertension and spontaneous hypokalemia (84.1%), while only 17.1% and 35.4% of centers screen patients with grade 2–3 hypertension. Screening rate is lower for cardiologists compared to other specialists. The main barriers to wider testing were challenges in interpreting the aldosterone/renin ratio under interfering medications and switching to non-interfering drugs. Clinical scores to predict the likelihood of PA and the definition of Standard Operating Procedures were identified as potential tools to boost screening rates. Testing for PHEO was mostly conducted in patients with typical symptoms (75.6%) and/or hypertensive crisis (74.4%). Only 37.8% of centers screened all patients with adrenal incidentaloma.

**Conclusion:**

This study highlights significant gaps in the screening and diagnosis of PA and PHEO across Italian centers and underscores the need for widespread and standardized diagnostic protocols.

**Supplementary Information:**

The online version contains supplementary material available at 10.1007/s40618-025-02532-5.

## Introduction

According to the Lancet Commission on Hypertension, one of the key actions to reduce the impact of high blood pressure globally is to increase the rate of diagnosis of secondary hypertension [1], whose prevalence, among the general hypertensive population, can be as high as 15–20%.

Primary aldosteronism (PA) is the most common endocrine cause of arterial hypertension: its prevalence ranges between 4% and 10% in primary care [2, 3] and increases among subjects with resistant hypertension [4, 5] or selected populations, such as patients with hypertension and hypokalaemia [6]. Compared to matched patients with essential hypertension, subjects affected by PA display higher rates of organ damage (i.e., microalbuminuria and left ventricular hypertrophy) and cardiovascular events, including atrial fibrillation, myocardial infarction, stroke, and heart failure [7, 8]. For this reason, a timely diagnosis and a targeted treatment are necessary to reduce the risk excess observed in patients with PA [9]. Despite its prevalence and guidelines recommendations [10, 11], the screening rate of PA is disappointingly low, even among patients with resistant hypertension or hypokalaemia [12, 13].

On the other hand, pheochromocytomas (PHEO) and paragangliomas are a rare cause of secondary hypertension, with an estimated prevalence in hypertensive patients varying between 0.2 and 0.6% [14, 15]. PHEO and paragangliomas are neuroendocrine tumors that arise from neural crest-derived cells of the adrenal medulla (pheochromocytoma) or the sympathetic or parasympathetic paraganglia and commonly produce one or more catecholamines [15]. Due to the broad spectrum of potential presenting symptoms, the diagnosis is often a challenge for clinicians and a median delay to diagnosis of about three years has been reported [16]. Hypertension (permanent or in the form of paroxysmal hypertensive crisis) is the most common clinical clue to suspect it [17], however nowadays in nearly two-thirds of the cases PHEOs are discovered as incidentalomas, rather than as a consequence of catecholamines’ excess related symptoms [18, 19]. The circulating catecholamines’ excess can result in life-threatening cardiovascular events, with nearly one third of the patients experiencing a cardiovascular complication [20]. This highlights the necessity of a timely diagnosis as well as a multidisciplinary team of experts in specialized centers for the management and treatment of these patients. The only curative treatment is surgical resection, that can also prevent progression to metastatic disease in case of PHEO with malignant potential [15].

The aim of this survey was to assess the diagnostic approach to these conditions among Italian Clinical Centers belonging to European and Italian Societies of Hypertension.

## Methods

### Study protocol

Between July and December 2023, we conducted a survey targeting physicians who treat patients with secondary hypertension, with the aim to assess the diagnosis of PA and PHEO. The survey consisted of a 10-item questionnaire that was administered through an online-form and via institutional communication channels of the Italian Society of Hypertension, to experts from 82 different centers (belonging to European and Italian Societies of Hypertension) across 14 Italian regions and to cardiologists from the ARCA (Associazioni Regionali Cardiologi Ambulatoriali) in Piedmont. The distribution of specialties represented in the survey reflects the composition of the Italian Society of Hypertension, which predominantly includes specialists in internal medicine, along with endocrinologists, nephrologists and geriatricians. All physicians who completed the questionnaire were included in the study, regardless of their medical specialty. For statistical purposes, all specialists other than internists and cardiologists were grouped into the category “Others.” Additionally, the participating centers were categorized into two main subgroups: excellence centers (recognized by the Societies as reference centers for hypertension care) and non-excellence centers.

### Statistical analysis

Data were analyzed using IBM SPSS Statistics 26 (IBM Corp, Armonk, NY) and GraphPad Prism 9.0 (GraphPad, La Jolla, CA). Sub-analyses were performed after stratification for geographical area (Northern Italy vs. Central-Southern Italy), specialty of responding physicians (Internal Medicine, Cardiology, or others), and center category (excellence vs. non-excellence centers). Data are reported as median and [interquartile range], or absolute number and (frequencies), respectively for scalar or ordinal variables. Groups were compared by Kruskal-Wallis or Mann-Whitney non-parametric tests, and Chi-square or Fisher’s tests, as appropriated. A p-value (*p*) of less than 0.05 was considered statistically significant.

## Results

### Characteristics of participating centers

**Fig. 1:**
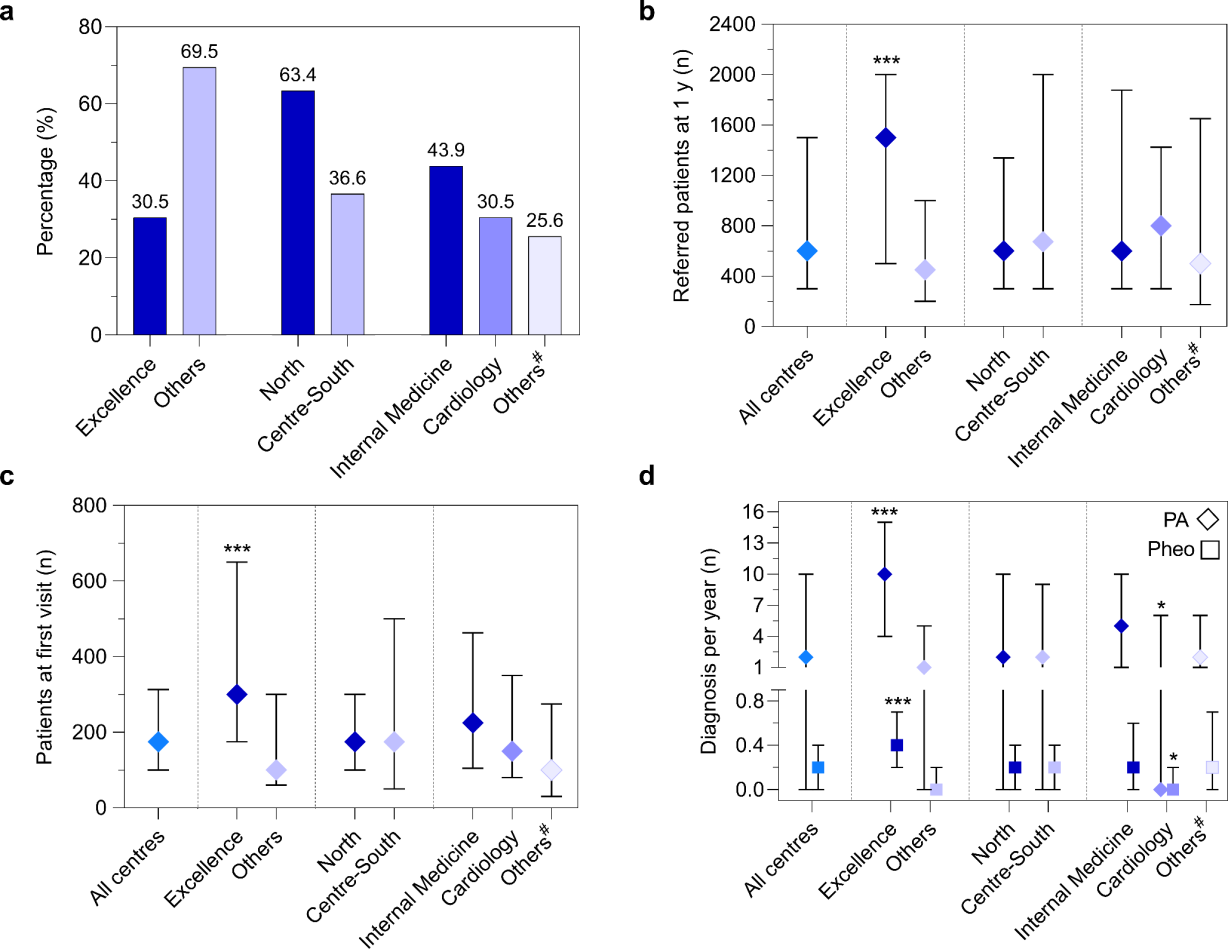
Characteristics, median number of referred patients and diagnosis of PA and PHEO of participating centers. (**a**) Stratification of centers by type, geographical area, and prevalent specialty. (**b**) Number of referred patients in 1 year. (**c**) Number of first visits. (**d**) Number of diagnosis of PA and PHEO per year in the last 5 years. Histograms show percentages; dot plots show median and interquartile range. #Others: endocrinology, nephrology and geriatrics. *p < 0.05; *** *p* < 0.001

A total of 82 centers completed a 10-item questionnaire (Table 1). Most of participating centers was located in northern Italy (63.4%) and one third was classified as Excellence center (30.5%). Various medical specialties took part in the investigation with Internal Medicine (43.9%) and Cardiology (30.5%) being the most prevalent. Endocrinologists represented 9.7% of the total responders (*n* = 8) and together with nephrologists and geriatricians were grouped into a miscellaneous category labelled as “Others” (25.6%) for comparisons purpose (Fig. 1a).

The median number of patients referred per center in one year was 600, with 175 patients evaluated at their first visit; 10% of subjects displayed resistant hypertension. Over the previous 5 years, 2444 cases of PA and 188 cases of PHEO were diagnosed, corresponding to a median of 2 PA cases and 0.2 PHEO per year per center (Supplementary Table 1).

As expected, excellence centers displayed a higher number of referred patients (1500 *versus* 450, *p* < 0.001; Fig. 1b), a higher number of first visits (300 *versus* 100, *p* < 0.001; Fig. 1c), with a higher median number of PA and PHEO diagnosis per year (10 PA cases and 0.4 PHEO cases) (Fig. 1d). A higher proportion of patients affected by resistant hypertension was reported by excellence centers compared to non-excellence centers, despite not statistically significant (15% *versus* 10%, *p* = 0.895). No differences were observed when stratified for geographical area (North *versus* Center-South), whereas in the subgroup analysis by specialty, cardiologists displayed a lower proportion of patients with resistant hypertension (10.0% *versus* 12.5% and 15.0%, respectively for Internal Medicine and other specialties; *p* = 0.048) as well as a lower number of PA and PHEO diagnosis per year in the last five years (Supplementary Table 1; Fig. 1d).

**Table 1:** Questionnaire on Diagnostic Approach of primary Aldosteronism and Pheochromocytoma

Survey on Diagnostic Approach of Primary Aldosteronism and Pheochromocytoma
*(1) What is your specialty?* a) Internal Medicine b) Nephrology c) Cardiology d) Endocrinology e) Other (specify)
*(2) What is the average number of patients with hypertension seen in your service/center in 1 year?* N = …
*(3) How many patients are seen as first visit?* N = …
*(4) What is the percentage of patients with resistant hypertension seen in your service/center?*
*(5) How many cases of PA were diagnosed per year on average in the last 5 years?* N = …
*(6) Which of the following patients*,* younger than 65 years*,* do you screen for PA with ARR?* *(you can indicate more than one category)* a) All patients with hypertension b) Patients with hypertension grade 2 c) Patients with hypertension grade 3 d) Patients with resistant hypertension e) Patients with hypertension and spontaneous hypokalemia f) Patients with hypertension and diuretic-induced hypokalemia g) Patients with hypertension and adrenal incidentaloma h) Patients with hypertension and family history of PA
*(7) In your daily practice*,* what are the obstacles to the frequent use of the ARR screening test in a patient with* *hypertension? (you can indicate more than one category)* a) Costs of the exam b) Difficulty in the interpretation of the ARR during interfering medications c) Difficulty to switch an interfering therapy to one without an effect d) Difficulty to perform a confirmatory test in case of a positive ARR e) Subtype diagnosis is invasive (AVS) and MRAs carry side effects f) Rarity of the disease (futility of the screening in most cases)
*(8) Which of the following could be useful in increasing the use of the ARR as screening test?* *(you can indicate more than one category)* a) None, I perform the test when necessary b) A clinical score which indicates the probability of a patient having this disease c) Having help with the interpretation of ARR results in patients with interfering medication d) It’s a rare disease and increasing screening is unnecessary e) The definition of a Standard Operating Procedure (SOP) in our scientific society
*(9) How many cases of Pheochromocytoma did you diagnosed in the last 5 years?* N = …
*(10) Which of the following patients do you test for Pheochromocytoma with plasma or urinary fractionated* *metanephrines? (you can indicate more than one category)* a) All patients with hypertension b) Patients with hypertension and tachycardia, sweating and headache c) Patients with hypertensive crises d) Patients with crises induced by steroids, opioids, or beta-blockers e) Patients with resistant hypertension f) Patients with hypertension and adrenal incidentaloma g) All patients with adrenal incidentaloma irrespective of blood pressure levels

The table reports the 10 items of the questionnaire to evaluate the diagnostic approach to Primary Aldosteronism and Pheochromocytoma

## Diagnosis of primary aldosteronism

**Fig. 2:**
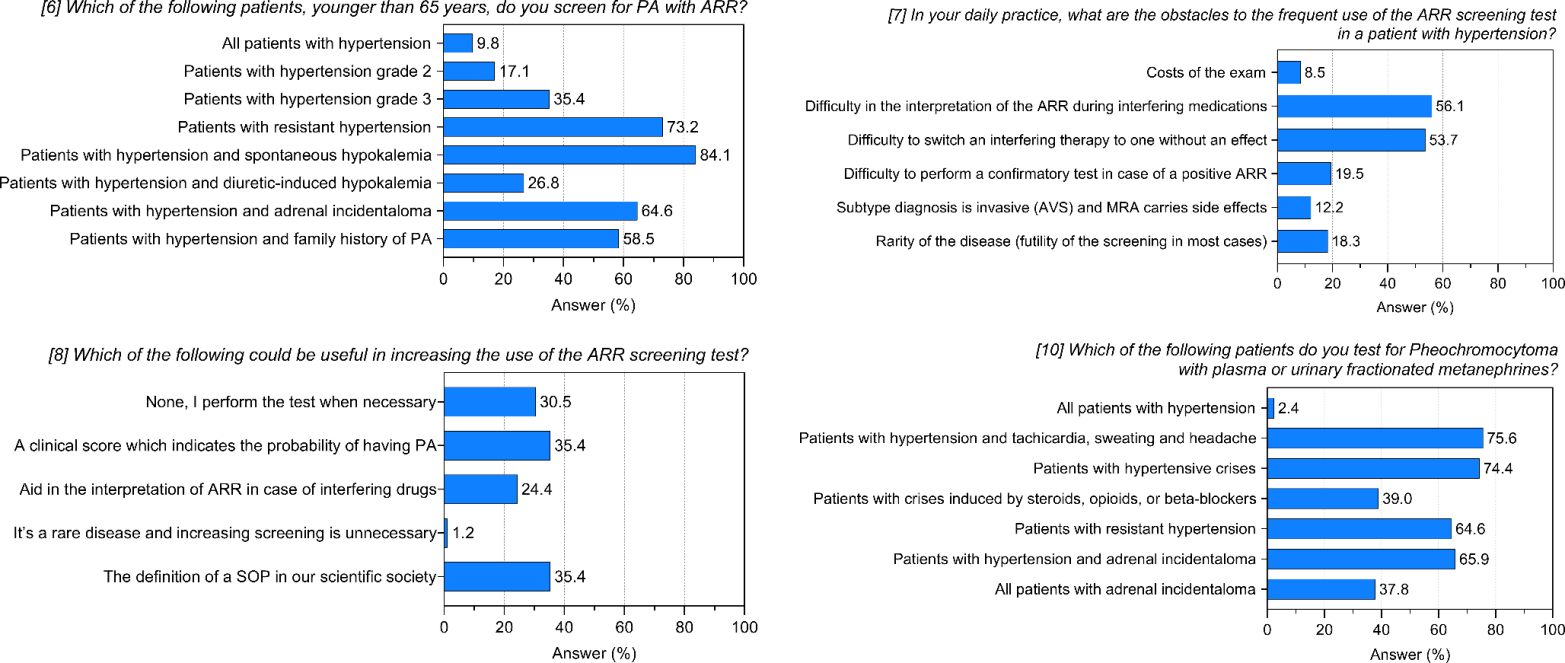
Answers to questions 6–8 and 10 of the questionnaire. Histograms show frequencies related to the 82 participating centers

Items 6, 7 and 8 of the questionnaire explored screening practices of centers (Supplementary Table 2; Fig. 2). Considering subjects younger than 65, most centers (84.1%) screen patients with hypertension and spontaneous hypokalemia, while only a small proportion (26.8%) screen those with diuretic-induced hypokalemia. A large part of the centers performs PA screening in presence of resistant hypertension (73.2%). Despite guidelines recommendations [10, 11], only 35.4 and 17.1% of centers consider ARR measurement in patients with grade 3 or 2 hypertension, respectively. More than half of the centers performs ARR when an adrenal incidentaloma is detected in hypertensive patients (64.6%) and about 60% considers PA screening in individuals with hypertension and family history of PA.

According to responding centers, the main barriers to using ARR for PA screening are its interpretation when measured under interfering medications (56.1%) and switching to non-interfering treatments (53.7%). Other reported issues included difficulty to perform a confirmatory test (19.5%) and concerns about invasiveness of subtype diagnosis with adrenal vein sampling and the side effects of MRAs (12.2%). Surprisingly, 20% of centers considered PA a rare disease, deeming screening unnecessary.

To address issues on PA screening, a clinical score to predict the probability of having PA and the definition of a Standard Operating Procedure (SOP) were the potential preferred tools (35.4% of responding centers). Help with ARR interpretation in the presence of interfering medications could be useful for 24.4% of the centers, while one center reported that screening for PA is unnecessary due to the rarity of the condition.

**Fig. 3:**
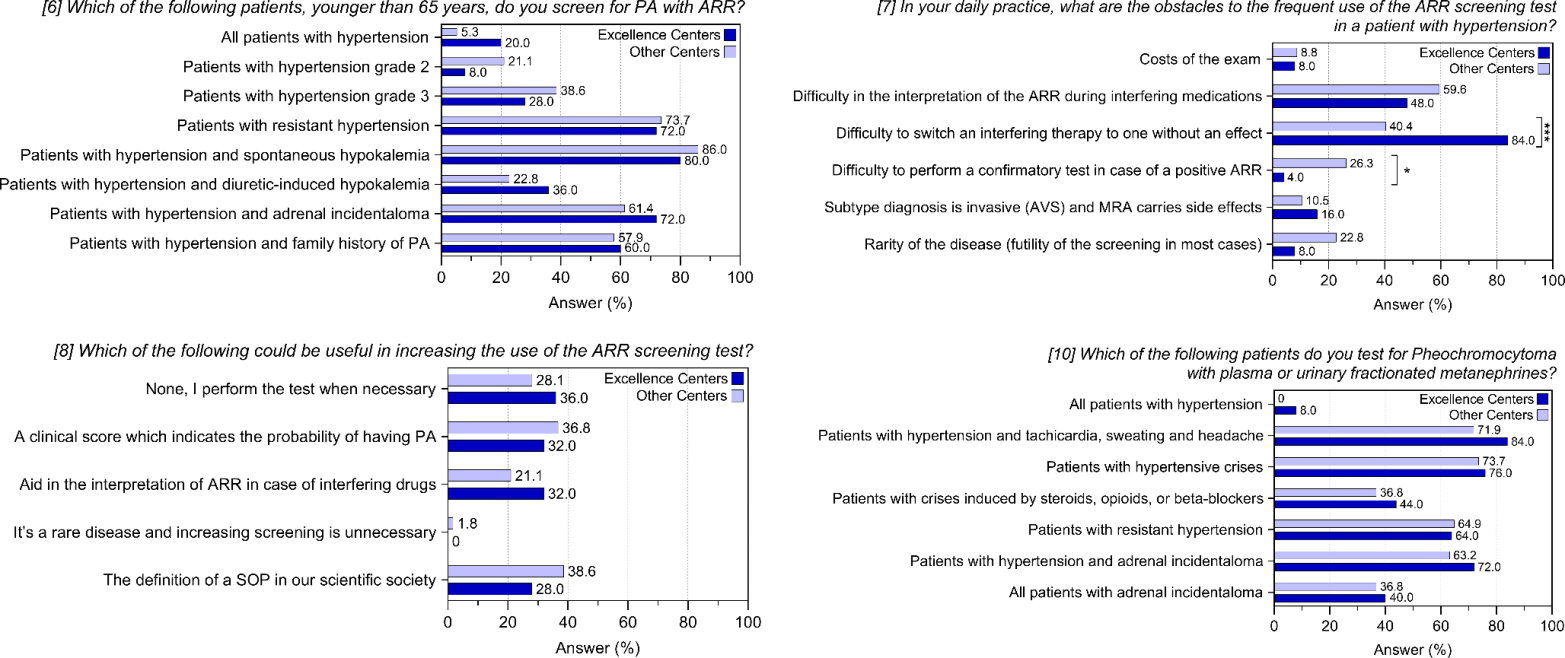
Answers to questions 6–8 and 10 of the questionnaire. Histograms show frequencies related to the participating centers after stratification for type of center (excellence *versus* non-excellence centers). *p < 0.05; ***p < 0.001

We then compared excellence centers with non-excellence centers (Supplementary Table 3; Fig. 3). No differences were observed in the screening of sub-populations (item 6); a larger proportion of excellence centers finds difficult to switch to non-interfering medication (84% *versus* 40.4%, *p* < 0.001), while on the other hand, non-excellence centers have more difficulties to perform a confirmatory test (26.3% *versus* 4%, *p* = 0.031; item 7). No differences were observed for the suggested measures to increase the use of ARR (item 8).

**Fig. 4:**
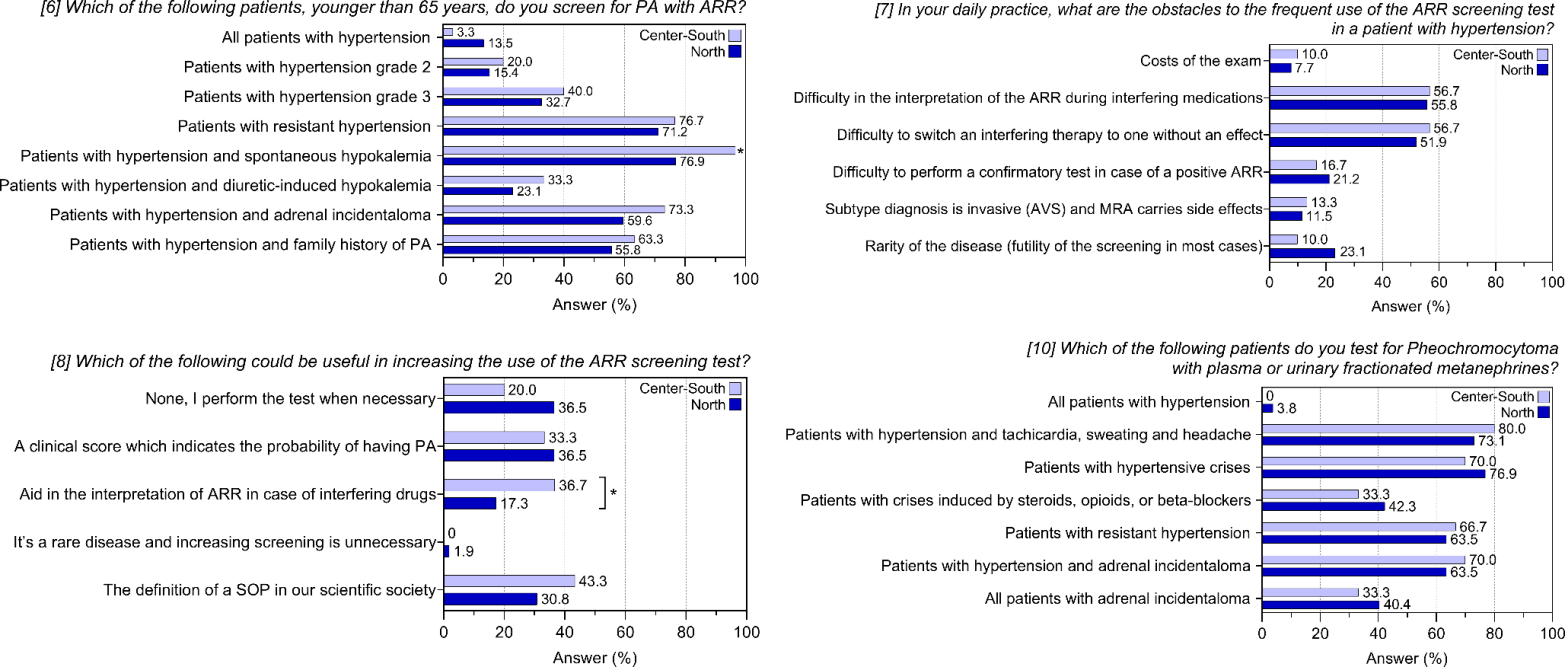
Answers to questions 6–8 and 10 of the questionnaire. Histograms show frequencies related to the participating centers after stratification for geographical area (North versus Center-South of Italy). *p < 0.05

The sub analysis by geographical area (Supplementary Table 4; Fig. 4) showed that a larger proportion of physicians from the central-southern Italy would screen patients younger than 65 with hypertension and spontaneous hypokalemia (96.7% *versus* 76.9%, *p* = 0.026; item 6). No differences were observed for item 7. More centers from the central-southern Italy would find useful some help with ARR interpretation when performed under interfering medications (36.7% *versus* 17.3%, *p* = 0.049; item 8).

**Fig. 5:**
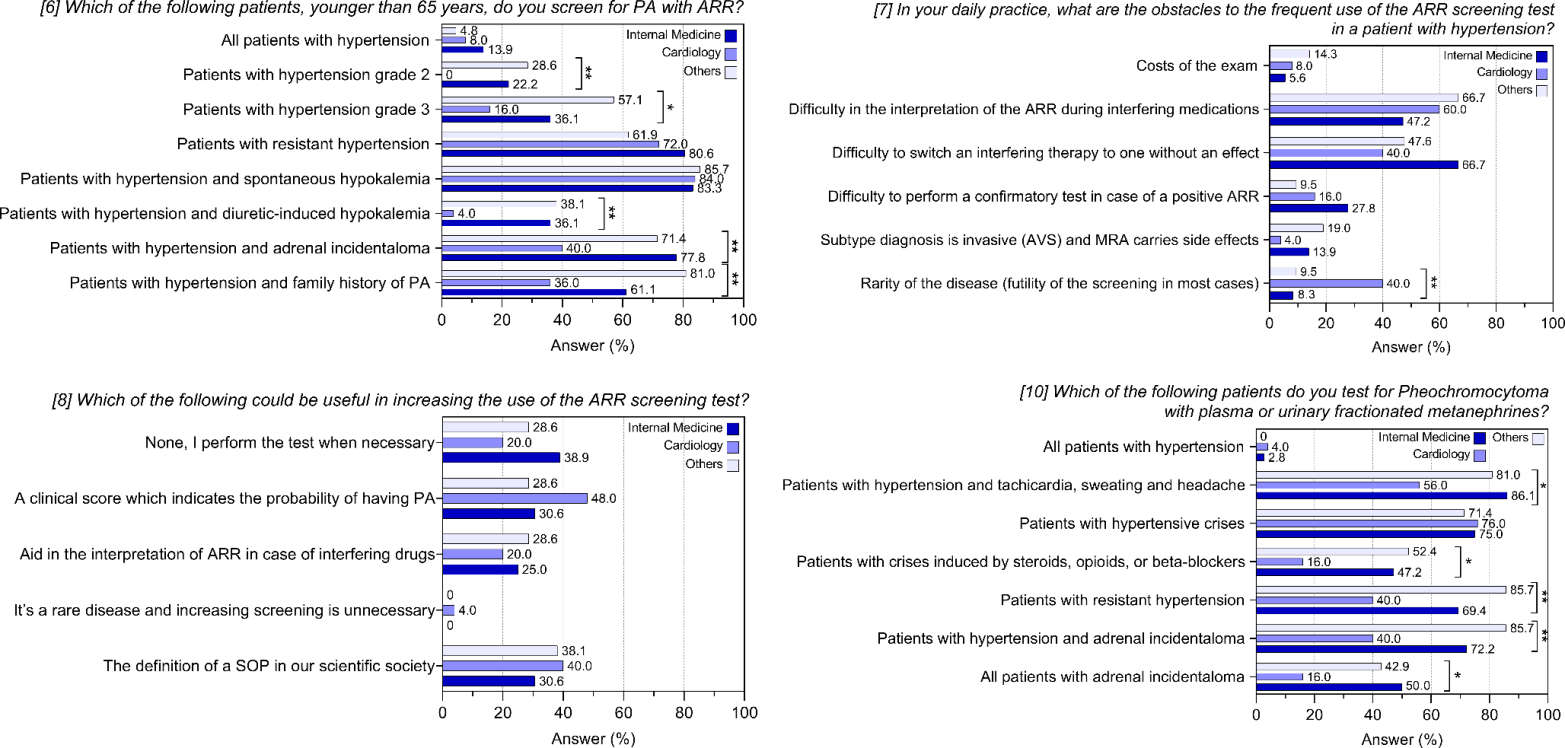
Answers to questions 6–8 and 10 of the questionnaire. Histograms show frequencies related to the participating centers after stratification for prevalent specialty (Internal Medicine versus Cardiology versus Others; “others” refers to endocrinology, nephrology and geriatrics). *p < 0.05; **p < 0.01

After stratification for specialty (Supplementary Table 5; Fig. 5), main differences were observed for cardiology as compared to internal medicine and other included specialties. Cardiologists display lower screening rates in patients with hypertension grade 2 or 3, diuretic-induced hypokalemia, adrenal incidentaloma, or family history of PA (*p* < 0.05 for all comparisons; item 6). A notable proportion of them (40%) considers PA a rare disease and then unnecessary to screen for (*p* = 0.006; item 7). No differences were observed in item 8. On the contrary, endocrinologists display a higher screening rate for almost all the categories of patients (Supplementary Table 6), particularly pronounced for patients with hypertension grade 3 (overall *p* = 0.003 and *p* = 0.015 compared to internists).

### Diagnosis of pheochromocytoma

Item 10 explored centers' approaches to PHEO screening using plasma or urinary fractionated metanephrines (Supplementary Table 2; Fig. 2): three-quarters of the centers perform PHEO biochemical tests in the presence of hypertension and tachycardia, sweating and headache (75.6%) and in case of hypertensive crises (74.4%). When crises are induced by steroids, opioids or beta-blockers, only 39% of the centers performs biochemical screening for PHEO. A significant proportion of participating centers considers screening for PHEO in patients with resistant hypertension (64.6%) and in patients with hypertension and adrenal incidentaloma (65.9%). Less than half of the centers (37.8%) screen all patients with an adrenal incidentaloma, irrespective of blood pressure levels. Finally, two out of 82 centers measure plasma or urinary fractionated metanephrines in all patients with hypertension.

No significant differences were found either in the comparison between excellence and non-excellence centers (Supplementary Table 3; Fig. 3) or in the comparison between different parts of Italy (Supplementary Table 4; Fig. 4).

The sub-analysis on specialty (Supplementary Table 5; Fig. 5) showed that the screening rate for PHEO is lower for cardiologists and higher for endocrinologists, who more frequently than all the other responders would test for PHEO all patients with an adrenal incidentaloma, irrespective of blood pressure values, and patients with resistant hypertension (even if no significant differences were found when comparing endocrinologists with internists; data not shown).

## Discussion

This study provides the results of a survey aimed to assess the diagnostic approach to PA and PHEO among 82 Italian centers belonging to the Italian and European Societies of Hypertension, across the whole country in a real-life setting.

Compared with the guidelines recommendations, significant gaps in screening rates of both diseases, particularly among cardiologists [10, 11, 15, 21], were identified. One of the most striking findings is the low annual rate of diagnosis of PA, with centers reporting an average of only 2 cases of PA annually. This is consistent with previous literature showing that screening rates for PA are low at both population and center level. In a European survey conducted among general practitioners in Germany and Italy [22], the screening rate for PA was 8% and 7% respectively, with a consequent low prevalence (2% in Germany and 1% in Italy). Similarly, the screening rate for PA is less than 2% in both patients with apparent resistant hypertension [12] and patients with hypokalemia [13], which represent the two categories with the highest PA prevalence [6, 23]. According with our results, the screening test with ARR is particularly underused among patients with grade II hypertension and with diuretic induced hypokalemia, despite the current ESH and Endocrine Society recommendations [10, 11], further broadened by the recent ESC guidelines for the management of elevated blood pressure and hypertension, indicating that screening for PA should be considered for all adult patients with blood pressure values ≥ 140/90 mmHg [24].

The main barriers identified in this study to PA screening are the unawareness of its actual prevalence, the difficulty in switching patients to non-interfering drugs, as well as the challenge of ARR interpretation under interfering medications.

Dedicated educational initiatives have been shown to significantly increase active PA testing, resulting in much higher screening rates than those typically observed [25], therefore, such measures should be strongly encouraged. About interfering medications, it should be acknowledged that ARR can be confidently interpreted in most cases, even in presence of two or more antihypertensive drugs [26], if the withdrawal or the switch to a non-interfering treatment is not feasible. Therefore, the interpretation of ARR should not represent an issue to discourage clinicians from screening for PA.

The use of clinical prediction scores and the implementation of SOPs were identified as potential solutions to increase the screening rate. Recently, a clinical score (Score To Predict Primary Aldosteronism– SToPPA score) and a machine-learning algorithm have been shown to efficiently predict the probability of having PA in patients with hypertension, aiding clinicians in selecting patients for screening. The application of this score can potentially reduce the number of screening tests by one third, without missing patients with unilateral surgically curable PA [27].

According to the available literature, consultation with an endocrinologist or a nephrologist, but not with a cardiologist was associated with a higher likelihood of being screened [12]. The lower screening rates among cardiologists, compared to other specialists, could be attributed to a lack of specific training in endocrine hypertension or the perception that screening is outside their clinical competence. These findings suggest a need for cross-specialty educational initiatives and the integration of standardized screening protocols into routine practice.

Unlike PA, PHEO is a rare cause of endocrine hypertension, often looked for in presence of typical signs and symptoms, but rarely found in this category of patients. The estimated prevalence in patients with typical signs and symptoms of presumed catecholamine excess is comprised between 0.2% and 0.6%, while it increases to 4–7% among patients with adrenal incidentaloma [21, 28]. While three out of four of the responders declared to screen for PHEO patients with hypertension, tachycardia, sweating and headache or hypertensive crises, only 37.8% of them stated to measure metanephrines in patients with adrenal incidentaloma (irrespective of blood pressure values). According to the recent Consensus of the Working Group on Endocrine Hypertension of the European Society of Hypertension [15], in all patients with an adrenal incidentaloma with tumour density > 10 Hounsfield Units the screening for pheochromocytomas should be considered, independently from blood pressure values.

According to a recent Italian nationwide survey about the screening for hypercortisolism among patients affected by arterial hypertension [29], it was observed that also Cushing syndrome and Mild Autonomous Cortisol Secretion (MACS) are rarely screened, mainly due to poor knowledge of European recommendations and of the appropriate screening tests.

The main limitations of this study lie in the absence of an independent external revision of the self-reported data by each center and the underrepresentation of some specialties. In particular, endocrinologists, which are actively involved in the diagnosis and treatment of secondary hypertension, were inadequately represented and this could limit the generalizability of our findings.

In conclusion, awareness, and screening rates of secondary forms of arterial hypertension, including PA and PHEO, are unsatisfactory. Our data highlight how wide the gap between scientific societies guidelines and the everyday clinical practice is, even among excellence centers. Addressing these gaps through education, standardized protocols, and clinical prediction tools could improve the timely identification and management of these conditions, ultimately enhancing patient outcomes and reducing the burden of hypertension-related complications.

## Electronic supplementary material

Below is the link to the electronic supplementary material.

Supplementary Material 1

Supplementary Material

## Data Availability

Data relevant to the present study are included in this published article and its supplementary information file.
