# Supplementary material for: Screening of primary aldosteronism and pheochromocytoma among patients with hypertension: an Italian nationwide survey
Source: J Endocrinol Invest. 2025 Jan 18;48(5):1197–205. doi: 10.1007/s40618-025-02532-5 (PMC12049289; doi:10.1007/s40618-025-02532-5)
Supplement: Supplementary file 1 — Supplementary Material 1 [file 40618_2025_2532_MOESM1_ESM.docx]

**SUPPLEMENTARY INFORMATION**

**Screening of primary aldosteronism and pheochromocytoma among patients with hypertension: an Italian nationwide survey**

**JOURNAL OF ENDOCRINOLOGICAL INVESTIGATION**

Silvia Monticone^1^, Jessica Goi^1^, Jacopo Burrello^1^, Guido Di Dalmazi^2,3^, Arrigo F.G. Cicero^4^, Costantino Mancusi^5^, Elena Coletti Moia^6^, Guido Iaccarino^5^, Franco Veglio^1^, Claudio Borghi^4^, Maria L. Muiesan^7^, Claudio Ferri,^8^ Paolo Mulatero^1^, Italian Society of Arterial Hypertension (SIIA).

^1^ Division of Internal Medicine 4 and Hypertension Unit, Department of Medical Sciences, University of Torino, Torino, Italy

^2^Division of Endocrinology and Diabetes Prevention and Care, IRCCS Azienda Ospedaliero-Universitaria di Bologna, Bologna, Italy

^3^Department of Medical and Surgical Sciences (DIMEC), Alma Mater Studiorum University of Bologna, Bologna, Italy.

^4^ Hypertension and Cardiovascular Risk Research Unit, Medical and Surgery Sciences Department, Alma Mater Studiorum University of Bologna, Bologna, Italy

^5^ Department of Advanced Biomedical Sciences, Federico II University of Naples, Via S. Pansini 5, 18 80131 Napoli, Italy

^6^ ARCA (Associazioni Regionali Cardiologi Ambulatoriali) Piemonte

^7^ Department of Clinical and Experimental Sciences University of Brescia Italy

^8^ Department of Life, Health and Environmental Sciences University of L'Aquila Italy

**Corresponding author:** Prof. Silvia Monticone, Division of Internal Medicine 4, Department of Medical Sciences, University of Torino, Città della Salute e della Scienza di Torino, Corso Dogliotti 14, 10126 Torino, Italy. Telephone/Fax number: +39.011.633.6959 / +39.011.633.6931. E-mail: silvia.monticone@unito.it ORCID 0000-0002-7322-2005

**List of Content**

Supplementary Table 1 - Characteristics of participating centers

Supplementary Table 2 – Diagnostic Approach of Primary Aldosteronism and Pheochromocytoma

Supplementary Table 3 – Sub-analyses after stratification for excellence centers

Supplementary Table 4 – Sub-analyses after stratification for geographical area

Supplementary Table 5 – Sub-analyses after stratification for prevalent specialty

Supplementary Table 6 – Stratification for prevalent specialty (sub-analysis on Endocrinology)

**Supplementary Table 1 –** **Characteristics of participating centers**

| **Variable** | | **Centers** (n=82) | | | |
| --- | --- | --- | --- | --- | --- |
| Median number of referred patients in 1 year (n) | | 600 [300; 1500] | | | |
| Patients evaluated as first visit (n) | | 175 [100; 313] | | | |
| Patients with resistant hypertension (%) | | 10.0 [5.0; 25.0] | | | |
| Cases of PA diagnosed per year in the last 5 years (n) | | 2 [0; 10] | | | |
| Cases of Pheochromocytoma diagnosed in the last 5 years (n) | | 1 [0; 2] | | | |
| Cases of Pheochromocytoma diagnosed per year in the last 5 years (n) | | 0.2 [0.0; 0.4] | | | |
| Geographical areas:  North of Italy (n)  Center-South of Italy (n) | | 52 (63.4%)  30 (36.6%) | | | |
| Prevalent specialty:  Internal Medicine (n)  Cardiology (n)  Others* (n) | | 36 (43.9%)  25 (30.5%)  21 (25.6%) | | | |
| Excellence centers (n) | | 25 (30.5%) | | | |
| **Excellence Centers** | **Yes** (n=25) | | **No** (n=57) | | ***P-*value** |
| Median number of referred patients in 1 year (n) | 1500 [500; 2000] | | 450 [200; 1000] | | **<0.001** |
| Patients evaluated as first visit (n) | 300 [175; 650] | | 100 [60; 300] | | **<0.001** |
| Patients with resistant hypertension (%) | 15.0 [6.5; 30.0] | | 10.0 [5.0; 22.5] | | 0.895 |
| Cases of PA diagnosed per year in the last 5 years (n) | 10 [4; 15] | | 1 [0; 5] | | **<0.001** |
| Cases of Pheochromocytoma diagnosed in the last 5 years (n) | 2 [1; 4] | | 0 [0; 1] | | **<0.001** |
| Cases of Pheochromocytoma diagnosed per year in the last 5 years (n) | 0.4 [0.2; 0.7] | | 0.0 [0.0; 0.2] | | **<0.001** |
| **Geographical Areas** | **North** (n=52) | | **Center-South** (n=30) | | ***P-*value** |
| Median number of referred patients in 1 year (n) | 600 [300; 1338] | | 675 [300; 2000] | | 0.585 |
| Patients evaluated as first visit (n) | 175 [100; 300] | | 175 [50; 500] | | 0.828 |
| Patients with resistant hypertension (%) | 10.0 [5.0; 28.8] | | 12.5 [5.0; 20.0] | | 0.919 |
| Cases of PA diagnosed per year in the last 5 years (n) | 2 [0; 10] | | 2 [0; 9] | | 0.661 |
| Cases of Pheochromocytoma diagnosed in the last 5 years (n) | 1 [0; 2] | | 1 [0; 2] | | 0.747 |
| Cases of Pheochromocytoma diagnosed per year in the last 5 years (n) | 0.2 [0.0; 0.4] | | 0.2 [0.0; 0.4] | | 0.747 |
| **Prevalent Specialty** | **Internal Medicine**  (n=36) | | **Cardiology**  (n=25) | **Others***  (n=21) | ***P-*value** |
| Median number of referred patients in 1 year (n) | 600 [300; 1876] | | 800 [300; 1425] | 500 [175; 1650] | 0.731 |
| Patients evaluated as first visit (n) | 225 [105; 463] | | 150 [80; 350] | 100 [30; 275] | 0.064 |
| Patients with resistant hypertension (%) | 12.5 [6.3; 30.0] | | 10.0 [5.0; 12.5] | 15.0 [10.0; 45.0] | **0.048** |
| Cases of PA diagnosed per year in the last 5 years (n) | 5 [1; 10] | | 0 [0; 6] | 2 [1; 6] | **0.015** |
| Cases of Pheochromocytoma diagnosed in the last 5 years (n) | 1 [0; 3] | | 0 [0; 1] | 1 [0; 4] | **0.027** |
| Cases of Pheochromocytoma diagnosed per year in the last 5 years (n) | 0.2 [0.0; 0.6] | | 0.0 [0.0; 0.2] | 0.2 [0.0; 0.7] | **0.027** |

The table reports characteristics, median number of referred patients and diagnosis of PA and PHEO of all the centers involved in the survey and after stratification for type of center (excellence *versus* non-excellence centers), geographical areas and prevalent specialty. Data are reported as median [interquartile range], or absolute number (percentage), as appropriated. A *P*-value less than 0.05 was considered significant and reported in bold characters. *Others refers to: endocrinology, nephrology and geriatrics.

**Supplementary Table 2 – Diagnostic Approach of Primary Aldosteronism and Pheochromocytoma**

| **All Centers** (n=82) |  |
| --- | --- |
| *(6) Which of the following patients, younger than 65 years, do you screen for PA with ARR?* | |
| 1. All patients with hypertension 2. Patients with hypertension grade 2 3. Patients with hypertension grade 3 4. Patients with resistant hypertension 5. Patients with hypertension and spontaneous hypokalemia 6. Patients with hypertension and diuretic-induced hypokalemia 7. Patients with hypertension and adrenal incidentaloma 8. Patients with hypertension and familial history of PA | 8 (9.8)  14 (17.1)  29 (35.4)  60 (73.2)  69 (84.1)  22 (26.8)  53 (64.6)  48 (58.5) |
| *(7) In your daily practice, what are the obstacles to the frequent use of the ARR screening test in a patient with hypertension?* | |
| 1. Costs of the exam 2. Difficulty in the interpretation of the ARR during interfering medications 3. Difficulty to switch an interfering therapy to one without an effect 4. Difficulty to perform a confirmatory test in case of a positive ARR 5. Subtype diagnosis is invasive (AVS) and MRA carries side effects 6. Rarity of the disease (futility of the screening in most cases) | 7 (8.5)  46 (56.1)  44 (53.7)  16 (19.5)  10 (12.2)  15 (18.3) |
| *(8) Which of the following could be useful in increasing the use of the ARR screening test?* | |
| 1. None, I perform the test when necessary 2. A clinical score which indicates the probability of a patient having this disease 3. Having help with the interpretation of ARR results in patients with interfering medication 4. It’s a rare disease and increasing screening is unnecessary 5. The definition of a SOP in our scientific society | 25 (30.5)  29 (35.4)  20 (24.4)  1 (1.2)  29 (35.4) |
| *(10) Which of the following patients do you screen for Pheochromocytoma with plasma or urinary fractionated metanephrines?* | |
| 1. All patients with hypertension 2. Patients with hypertension and tachycardia, sweating and headache 3. Patients with hypertensive crises 4. Patients with crises induced by steroids, opioids, or beta-blockers 5. Patients with resistant hypertension 6. Patients with hypertension and adrenal incidentaloma 7. All patients with adrenal incidentaloma irrespective of blood pressure levels | 2 (2.4)  62 (75.6)  61 (74.4)  32 (39.0)  53 (64.6)  54 (65.9)  31 (37.8) |

Answers to questions 6-8 and 10 of the questionnaire. Data are reported as absolute numbers and frequencies related to the 82 participating centers.

**Supplementary Table 3 – Sub-analyses after stratification for excellence centers**

| **Excellence Centers** | **Yes**  (n=25) | **No**  (n=57) | ***P-*value** |
| --- | --- | --- | --- |
| *(6) Which of the following patients, younger than 65 years, do you screen for PA with ARR?* | | | |
| 1. All patients with hypertension 2. Patients with hypertension grade 2 3. Patients with hypertension grade 3 4. Patients with resistant hypertension 5. Patients with hypertension and spontaneous hypokalemia 6. Patients with hypertension and diuretic-induced hypokalemia 7. Patients with hypertension and adrenal incidentaloma 8. Patients with hypertension and familial history of PA | 5 (20.0)  2 (8.0)  7 (28.0)  18 (72.0)  20 (80.0)  9 (36.0)  18 (72.0)  15 (60.0) | 3 (5.3)  12 (21.1)  22 (38.6)  42 (73.7)  49 (86.0)  13 (22.8)  35 (61.4)  33 (57.9) | 0.052  0.208  0.357  0.862  0.522  0.215  0.357  0.862 |
| *(7) In your daily practice, what are the obstacles to the frequent use of the ARR screening test in a patient with hypertension?* | | | |
| 1. Costs of the exam 2. Difficulty in the interpretation of the ARR during interfering medications 3. Difficulty to switch an interfering therapy to one without an effect 4. Difficulty to perform a confirmatory test in case of a positive ARR 5. Subtype diagnosis is invasive (AVS) and MRA carries side effects 6. Rarity of the disease (futility of the screening in most cases) | 2 (8.0)  12 (48.0)  21 (84.0)  1 (4.0)  4 (16.0)  2 (8.0) | 5 (8.8)  34 (59.6)  23 (40.4)  15 (26.3)  6 (10.5)  13 (22.8) | 1.000  0.327  **<0.001**  **0.031**  0.716  0.133 |
| *(8) Which of the following could be useful in increasing the use of the ARR screening test?* | | | |
| 1. None, I perform the test when necessary 2. A clinical score which indicates the probability of a patient having this disease 3. Having help with the interpretation of ARR results in patients with interfering medication 4. It’s a rare disease and increasing screening is unnecessary 5. The definition of a SOP in our scientific society | 9 (36.0)  8 (32.0)  8 (32.0)  0 (0.0)  7 (28.0) | 16 (28.1)  21 (36.8)  12 (21.1)  1 (1.8)  22 (38.6) | 0.471  0.671  0.288  1.000  0.357 |
| *(10) Which of the following patients do you screen for Pheochromocytoma with plasma or urinary fractionated metanephrines?* | | | |
| 1. All patients with hypertension 2. Patients with hypertension and tachycardia, sweating and headache 3. Patients with hypertensive crises 4. Patients with crises induced by steroids, opioids, or beta-blockers 5. Patients with resistant hypertension 6. Patients with hypertension and adrenal incidentaloma 7. All patients with adrenal incidentaloma irrespective of blood pressure levels | 2 (8.0)  21 (84.0)  19 (76.0)  11 (44.0)  16 (64.0)  18 (72.0)  10 (40.0) | 0 (0.0)  41 (71.9)  42 (73.7)  21 (36.8)  37 (64.9)  36 (63.2)  21 (36.8) | 0.090  0.279  0.823  0.543  0.920  0.439  0.791 |

Responses to questions 6-8 and 10 of the questionnaire after stratification for type of center. Data are reported as absolute numbers and frequencies. A *P*-value less than 0.05 was considered significant and reported in bold characters.

**Supplementary Table 4 – Sub-analyses after stratification for geographical area**

| **Geographical Area** | **North**  (n=52) | **Center-South**  (n=30) | ***P-*value** |
| --- | --- | --- | --- |
| *(6) Which of the following patients, younger than 65 years, do you screen for PA with ARR?* | | | |
| 1. All patients with hypertension 2. Patients with hypertension grade 2 3. Patients with hypertension grade 3 4. Patients with resistant hypertension 5. Patients with hypertension and spontaneous hypokalemia 6. Patients with hypertension and diuretic-induced hypokalemia 7. Patients with hypertension and adrenal incidentaloma 8. Patients with hypertension and familial history of PA | 7 (13.5)  8 (15.4)  17 (32.7)  37 (71.2)  40 (76.9)  12 (23.1)  31 (59.6)  29 (55.8) | 1 (3.3)  6 (20.0)  12 (40.0)  23 (76.7)  29 (96.7)  10 (33.3)  22 (73.3)  19 (63.3) | 0.247  0.762  0.632  0.618  **0.026**  0.313  0.210  0.502 |
| *(7) In your daily practice, what are the obstacles to the frequent use of the ARR screening test in a patient with hypertension?* | | | |
| 1. Costs of the exam 2. Difficulty in the interpretation of the ARR during interfering medications 3. Difficulty to switch an interfering therapy to one without an effect 4. Difficulty to perform a confirmatory test in case of a positive ARR 5. Subtype diagnosis is invasive (AVS) and MRA carries side effects 6. Rarity of the disease (futility of the screening in most cases) | 4 (7.7)  29 (55.8)  27 (51.9)  11 (21.2)  6 (11.5)  12 (23.1) | 3 (10.0)  17 (56.7)  17 (56.7)  5 (16.7)  4 (13.3)  3 (10.0) | 1.000  0.920  0.680  0.775  1.000  0.235 |
| *(8) Which of the following could be useful in increasing the use of the ARR screening test?* | | | |
| 1. None, I perform the test when necessary 2. A clinical score which indicates the probability of a patient having this disease 3. Having help with the interpretation of ARR results in patients with interfering medication 4. It’s a rare disease and increasing screening is unnecessary 5. The definition of a SOP in our scientific society | 19 (36.5)  19 (36.5)  9 (17.3)  1 (1.9)  16 (30.8) | 6 (20.0)  10 (33.3)  11 (36.7)  0 (0.0)  13 (43.3) | 0.117  0.764  **0.049**  1.000  0.252 |
| *(10) Which of the following patients do you screen for Pheochromocytoma with plasma or urinary fractionated metanephrines?* | | | |
| 1. All patients with hypertension 2. Patients with hypertension and tachycardia, sweating and headache 3. Patients with hypertensive crises 4. Patients with crises induced by steroids, opioids, or beta-blockers 5. Patients with resistant hypertension 6. Patients with hypertension and adrenal incidentaloma 7. All patients with adrenal incidentaloma irrespective of blood pressure levels | 2 (3.8)  38 (73.1)  40 (76.9)  22 (42.3)  33 (63.5)  33 (63.5)  21 (40.4) | 0 (0.0)  24 (80.0)  21 (70.0)  10 (33.3)  20 (66.7)  21 (70.0)  10 (33.3) | 0.530  0.584  0.488  0.424  0.764  0.549  0.527 |

Answers to questions 6-8 and 10 of the questionnaire after stratification for geographical area. Data are reported as absolute numbers and frequencies. A *P*-value less than 0.05 was considered significant and reported in bold.

**Supplementary Table 5 – Sub-analyses after stratification for prevalent specialty**

| **Prevalent Specialty** | **Internal Medicine**  (n=36) | **Cardiology**  (n=25) | **Others***  (n=21) | ***P-*value** |
| --- | --- | --- | --- | --- |
| *(6) Which of the following patients, younger than 65 years, do you screen for PA with ARR?* | | | | |
| 1. All patients with hypertension 2. Patients with hypertension grade 2 3. Patients with hypertension grade 3 4. Patients with resistant hypertension 5. Patients with hypertension and spontaneous hypokalemia 6. Patients with hypertension and diuretic-induced hypokalemia 7. Patients with hypertension and adrenal incidentaloma 8. Patients with hypertension and familial history of PA | 5 (13.9)  8 (22.2)  13 (36.1)  29 (80.6)  30 (83.3)  13 (36.1)  28 (77.8)  22 (61.1) | 2 (8.0)  0 (0.0)  4 (16.0)  18 (72.0)  21 (84.0)  1 (4.0)  10 (40.0)  9 (36.0) | 1 (4.8)  6 (28.6)  12 (57.1)  13 (61.9)  18 (85.7)  8 (38.1)  15 (71.4)  17 (81.0) | 0.639  **0.008**  **0.015**  0.304  0.851  **0.004**  **0.008**  **0.009** |
| *(7) In your daily practice, what are the obstacles to the frequent use of the ARR screening test in a patient with hypertension?* | | | | |
| 1. Costs of the exam 2. Difficulty in the interpretation of the ARR during interfering medications 3. Difficulty to switch an interfering therapy to one without an effect 4. Difficulty to perform a confirmatory test in case of a positive ARR 5. Subtype diagnosis is invasive (AVS) and MRA carries side effects 6. Rarity of the disease (futility of the screening in most cases) | 2 (5.6)  17 (47.2)  24 (66.7)  10 (27.8)  5 (13.9)  3 (8.3) | 2 (8.0)  15 (60.0)  10 (40.0)  4 (16.0)  1 (4.0)  10 (40.0) | 3 (14.3)  14 (66.7)  10 (47.6)  2 (9.5)  4 (19.0)  2 (9.5) | 0.532  0.323  0.099  0.266  0.289  **0.006** |
| *(8) Which of the following could be useful in increasing the use of the ARR screening test?* | | | | |
| 1. None, I perform the test when necessary 2. A clinical score which indicates the probability of a patient having this disease 3. Having help with the interpretation of ARR results in patients with interfering medication 4. It’s a rare disease and increasing screening is unnecessary 5. The definition of a SOP in our scientific society | 14 (38.9)  11 (30.6)  9 (25.0)  0 (0.0)  11 (30.6) | 5 (20.0)  12 (48.0)  5 (20.0)  1 (4.0)  10 (40.0) | 6 (28.6)  6 (28.6)  6 (28.6)  0 (0.0)  8 (38.1) | 0.283  0.282  0.807  0.561  0.715 |
| *(10) Which of the following patients do you screen for Pheochromocytoma with plasma or urinary fractionated metanephrines?* | | | | |
| 1. All patients with hypertension 2. Patients with hypertension and tachycardia, sweating and headache 3. Patients with hypertensive crises 4. Patients with crises induced by steroids, opioids, or beta-blockers 5. Patients with resistant hypertension 6. Patients with hypertension and adrenal incidentaloma 7. All patients with adrenal incidentaloma irrespective of blood pressure levels | 1 (2.8)  31 (86.1)  27 (75.0)  17 (47.2)  25 (69.4)  26 (72.2)  18 (50.0) | 1 (4.0)  14 (56.0)  19 (76.0)  4 (16.0)  10 (40.0)  10 (40.0)  4 (16.0) | 0 (0.0)  17 (81.0)  15 (71.4)  11 (52.4)  18 (85.7)  18 (85.7)  9 (42.9) | 1.000  **0.025**  0.932  **0.014**  **0.004**  **0.003**  **0.018** |

Responses to questions 6-8 and 10 of the questionnaire after stratification according to prevalent specialty. Data are reported as absolute numbers and frequencies. A *P*-value less than 0.05 was considered significant and reported in bold. *Others refers to: endocrinology, nephrology and geriatrics.

**Supplementary Table 6 – Stratification for prevalent specialty (sub-analysis on Endocrinology)**

| **Prevalent Specialty** | **Internal Medicine**  (n=36) | **Cardiology**  (n=25) | **Endocrinology**  (n=8) | **Others***  (n=13) | ***P-*value** |
| --- | --- | --- | --- | --- | --- |
| *(6) Which of the following patients, younger than 65 years, do you screen for PA with ARR?* | | | | | |
| 1. All patients with hypertension 2. Patients with hypertension grade 2 3. Patients with hypertension grade 3 4. Patients with resistant hypertension 5. Patients with hypertension and spontaneous hypokalemia 6. Patients with hypertension and diuretic-induced hypokalemia 7. Patients with hypertension and adrenal incidentaloma 8. Patients with hypertension and family history of PA | 5 (13.9)  8 (22.2)  13 (36.1)  29 (80.6)  30 (83.3)  13 (36.1)  28 (77.8)  22 (61.1) | 2 (8.0)  0 (0.0)  4 (16.0)  18 (72.0)  21 (84.0)  1 (4.0)  10 (40.0)  9 (36.0) | 1 (12.5)  3 (37.5)  7 (87.5)  7 (87.5)  6 (75.0)  4 (50.0)  7 (87.5)  7 (87.5) | 0 (0.0)  3 (23.1)  5 (38.5)  6 (46.2)  12 (92.3)  4 (30.8)  8 (61.5)  10 (76.9) | 0.581  **0.011**  **0.003**  0.100  0.769  **0.005**  **0.011**  **0.021** |
| *(7) In your daily practice, what are the obstacles to the frequent use of the ARR screening test in a patient with hypertension?* | | | | | |
| 1. Costs of the exam 2. Difficulty in the interpretation of the ARR during interfering medications 3. Difficulty to switch an interfering therapy to one without an effect 4. Difficulty to perform a confirmatory test in case of a positive ARR 5. Subtype diagnosis is invasive (AVS) and MRA carries side effects 6. Rarity of the disease (futility of the screening in most cases) | 2 (5.6)  17 (47.2)  24 (66.7)  10 (27.8)  5 (13.9)  3 (8.3) | 2 (8.0)  15 (60.0)  10 (40.0)  4 (16.0)  1 (4.0)  10 (40.0) | 1 (12.5)  5 (62.5)  3 (37.5)  1 (12.5)  3 (37.5)  0 (0.0) | 2 (15.4)  9 (69.2)  7 (53.8)  1 (7.7)  1 (7.7)  2 (15.4) | 0.561  0.521  0.157  0.440  0.096  **0.010** |
| *(8) Which of the following could be useful in increasing the use of the ARR screening test?* | | | | | |
| 1. None, I perform the test when necessary 2. A clinical score which indicates the probability of a patient having this disease 3. Having help with the interpretation of ARR results in patients with interfering medication 4. It’s a rare disease and increasing screening is unnecessary 5. The definition of a SOP in our scientific society | 14 (38.9)  11 (30.6)  9 (25.0)  0 (0.0)  11 (30.6) | 5 (20.0)  12 (48.0)  5 (20.0)  1 (4.0)  10 (40.0) | 3 (37.5)  2 (25.0)  1 (12.5)  0 (0.0)  5 (62.5) | 3 (23.1)  4 (30.8)  5 (38.5)  0 (0.0)  3 (23.1) | 0.390  0.510  0.555  0.561  0.267 |
| *(10) Which of the following patients do you test for Pheochromocytoma with plasma or urinary fractionated metanephrines?* | | | | | |
| 1. All patients with hypertension 2. Patients with hypertension and tachycardia, sweating and headache 3. Patients with hypertensive crises 4. Patients with crises induced by steroids, opioids, or beta-blockers 5. Patients with resistant hypertension 6. Patients with hypertension and adrenal incidentaloma 7. All patients with adrenal incidentaloma irrespective of blood pressure levels | 1 (2.8)  31 (86.1)  27 (75.0)  17 (47.2)  25 (69.4)  26 (72.2)  18 (50.0) | 1 (4.0)  14 (56.0)  19 (76.0)  4 (16.0)  10 (40.0)  10 (40.0)  4 (16.0) | 0 (0.0)  7 (87.5)  8 (100.0)  5 (62.5)  7 (87.5)  7 (87.5)  6 (75.0) | 0 (0.0)  10 (76.9)  7 (53.8)  6 (46.2)  11 (84.6)  11 (84.6)  3 (23.1) | 1.000  0.053  0.137  **0.026**  **0.014**  **0.010**  **0.004** |

Answers to questions 6-8 and 10 after stratification for specialty, including Internal Medicine, Cardiology, Endocrinology and others. Data are reported as absolute number (frequency). A *P*-value less than 0.05 was considered significant and reported in bold. *Others refers to nephrology and geriatrics.
