# Supplementary material for: Screening of primary aldosteronism and pheochromocytoma among patients with hypertension: an Italian nationwide survey
Source: J Endocrinol Invest. 2025 Jan 18;48(5):1197–205. doi: 10.1007/s40618-025-02532-5 (PMC12049289; doi:10.1007/s40618-025-02532-5)
Supplement: Supplementary file 2 — Supplementary Material [file 40618_2025_2532_MOESM2_ESM.pdf]

SUPPLEMENTARY INFORMATION

Screening of primary aldosteronism and pheochromocytoma among patients with hypertension: an Italian nationwide survey

JOURNAL OF ENDOCRINOLOGICAL INVESTIGATION

Silvia Monticone[1], Jessica Goi[1], Jacopo Burrello[1], Guido Di Dalmazi[2,3], Arrigo F.G. Cicero[4], Costantino Mancusi[5], Elena Coletti Moia[6], Guido Iaccarino[5], Franco Veglio[1], Claudio Borghi[4], Maria L. Muiesan[7], Claudio Ferri[8], Paolo Mulatero[1], Italian Society of Arterial Hypertension (SIIA).

[1] Division of Internal Medicine 4 and Hypertension Unit, Department of Medical Sciences, University of Torino, Torino, Italy
[2]Division of Endocrinology and Diabetes Prevention and Care, IRCCS Azienda Ospedaliero-Universitaria di Bologna, Bologna, Italy
[3]Department of Medical and Surgical Sciences (DIMEC), Alma Mater Studiorum University of Bologna, Bologna, Italy.
[4] Hypertension and Cardiovascular Risk Research Unit, Medical and Surgery Sciences Department, Alma Mater Studiorum University of Bologna, Bologna, Italy
[5] Department of Advanced Biomedical Sciences, Federico II University of Naples, Via S. Pansini 5, 18 80131 Napoli, Italy
[6] ARCA (Associazioni Regionali Cardiologi Ambulatoriali) Piemonte
[7] Department of Clinical and Experimental Sciences University of Brescia Italy
[8] Department of Life, Health and Environmental Sciences University of L'Aquila Italy

Corresponding author: Prof. Silvia Monticone, Division of Internal Medicine 4, Department of Medical Sciences, University of Torino, Città della Salute e della Scienza di Torino, Corso Dogliotti 14, 10126 Torino, Italy. Telephone/Fax number: +39.011.633.6959 / +39.011.633.6931. E-mail: silvia.monticone@unito.it ORCID 0000-0002-7322-2005

List of Content

Supplementary Table 1 - Characteristics of participating centers
Supplementary Table 2 – Diagnostic Approach of Primary Aldosteronism and Pheochromocytoma
Supplementary Table 3 – Sub-analyses after stratification for excellence centers
Supplementary Table 4 – Sub-analyses after stratification for geographical area
Supplementary Table 5 – Sub-analyses after stratification for prevalent specialty
Supplementary Table 6 – Stratification for prevalent specialty (sub-analysis on Endocrinology)

Supplementary Table 1 – Characteristics of participating centers

**Variable**	**Centers** (n=82)		
Median number of referred patients in 1 year (n)	600 [300; 1500]		
Patients evaluated as first visit (n)	175 [100; 313]		
Patients with resistant hypertension (%)	10.0 [5.0; 25.0]		
Cases of PA diagnosed per year in the last 5 years (n)	2 [0; 10]		
Cases of Pheochromocytoma diagnosed in the last 5 years (n)	1 [0; 2]		
Cases of Pheochromocytoma diagnosed per year in the last 5 years (n)	0.2 [0.0; 0.4]		
Geographical areas:			
North of Italy (n)	52 (63.4%)		
Center-South of Italy (n)	30 (36.6%)		
Prevalent specialty:			
Internal Medicine (n)	36 (43.9%)		
Cardiology (n)	25 (30.5%)		
Others* (n)	21 (25.6%)		
Excellence centers (n)	25 (30.5%)		
**Excellence Centers**	**Yes** (n=25)	**No** (n=57)	***P*-value**
Median number of referred patients in 1 year (n)	1500 [500; 2000]	450 [200; 1000]	**<0.001**
Patients evaluated as first visit (n)	300 [175; 650]	100 [60; 300]	**<0.001**
Patients with resistant hypertension (%)	15.0 [6.5; 30.0]	10.0 [5.0; 22.5]	0.895
Cases of PA diagnosed per year in the last 5 years (n)	10 [4; 15]	1 [0; 5]	**<0.001**
Cases of Pheochromocytoma diagnosed in the last 5 years (n)	2 [1; 4]	0 [0; 1]	**<0.001**
Cases of Pheochromocytoma diagnosed per year in the last 5 years (n)	0.4 [0.2; 0.7]	0.0 [0.0; 0.2]	**<0.001**

**Geographical Areas**	**North** (n=52)		**Center-South** (n=30)	***P*-value**
Median number of referred patients in 1 year (n)	600 [300; 1338]		675 [300; 2000]	0.585
Patients evaluated as first visit (n)	175 [100; 300]		175 [50; 500]	0.828
Patients with resistant hypertension (%)	10.0 [5.0; 28.8]		12.5 [5.0; 20.0]	0.919
Cases of PA diagnosed per year in the last 5 years (n)	2 [0; 10]		2 [0; 9]	0.661
Cases of Pheochromocytoma diagnosed in the last 5 years (n)	1 [0; 2]		1 [0; 2]	0.747
Cases of Pheochromocytoma diagnosed per year in the last 5 years (n)	0.2 [0.0; 0.4]		0.2 [0.0; 0.4]	0.747
**Prevalent Specialty**	**Internal Medicine** (n=36)	**Cardiology** (n=25)	**Others*** (n=21)	***P*-value**
Median number of referred patients in 1 year (n)	600 [300; 1876]	800 [300; 1425]	500 [175; 1650]	0.731
Patients evaluated as first visit (n)	225 [105; 463]	150 [80; 350]	100 [30; 275]	0.064
Patients with resistant hypertension (%)	12.5 [6.3; 30.0]	10.0 [5.0; 12.5]	15.0 [10.0; 45.0]	**0.048**
Cases of PA diagnosed per year in the last 5 years (n)	5 [1; 10]	0 [0; 6]	2 [1; 6]	**0.015**
Cases of Pheochromocytoma diagnosed in the last 5 years (n)	1 [0; 3]	0 [0; 1]	1 [0; 4]	**0.027**
Cases of Pheochromocytoma diagnosed per year in the last 5 years (n)	0.2 [0.0; 0.6]	0.0 [0.0; 0.2]	0.2 [0.0; 0.7]	**0.027**

The table reports characteristics, median number of referred patients and diagnosis of PA and PHEO of all the centers involved in the survey and after stratification for type of center (excellence *versus* non-excellence centers), geographical areas and prevalent specialty. Data are reported as median [interquartile range], or absolute number (percentage), as appropriated. A *P*-value less than 0.05 was considered significant and reported in bold characters. *Others refers to: endocrinology, nephrology and geriatrics.

Supplementary Table 2 – Diagnostic Approach of Primary Aldosteronism and Pheochromocytoma

**All Centers** (n=82)	
(6) Which of the following patients, younger than 65 years, do you screen for PA with ARR?	
a) All patients with hypertension	8 (9.8)
b) Patients with hypertension grade 2	14 (17.1)
c) Patients with hypertension grade 3	29 (35.4)
d) Patients with resistant hypertension	60 (73.2)
e) Patients with hypertension and spontaneous hypokalemia	69 (84.1)
f) Patients with hypertension and diuretic-induced hypokalemia	22 (26.8)
g) Patients with hypertension and adrenal incidentaloma	53 (64.6)
h) Patients with hypertension and family history of PA	48 (58.5)
(7) In your daily practice, what are the obstacles to the frequent use of the ARR screening test in a patient with hypertension?	
a) Costs of the exam	7 (8.5)
b) Difficulty in the interpretation of the ARR during interfering medications	46 (56.1)
c) Difficulty to switch an interfering therapy to one without an effect	44 (53.7)
d) Difficulty to perform a confirmatory test in case of a positive ARR	16 (19.5)
e) Subtype diagnosis is invasive (AVS) and MRA carries side effects	10 (12.2)
f) Rarity of the disease (futility of the screening in most cases)	15 (18.3)
(8) Which of the following could be useful in increasing the use of the ARR screening test?	
a) None, I perform the test when necessary	25 (30.5)
b) A clinical score which indicates the probability of a patient having this disease	29 (35.4)
c) Having help with the interpretation of ARR results in patients with interfering medication	20 (24.4)
d) It's a rare disease and increasing screening is unnecessary	1 (1.2)
e) The definition of a SOP in our scientific society	29 (35.4)
(10) Which of the following patients do you test for Pheochromocytoma with plasma or urinary fractionated metanephrines?	
a) All patients with hypertension	2 (2.4)
b) Patients with hypertension and tachycardia, sweating and headache	62 (75.6)
c) Patients with hypertensive crises	61 (74.4)
d) Patients with crises induced by steroids, opioids, or beta-blockers	32 (39.0)
e) Patients with resistant hypertension	53 (64.6)
f) Patients with hypertension and adrenal incidentaloma	54 (65.9)
g) All patients with adrenal incidentaloma irrespective of blood pressure levels	31 (37.8)

Answers to questions 6-8 and 10 of the questionnaire. Data are reported as absolute numbers and frequencies related to the 82 participating centers.

Supplementary Table 3 – Sub-analyses after stratification for excellence centers

**Excellence Centers**	**Yes** (n=25)	**No** (n=57)	***P*-value**
(6) Which of the following patients, younger than 65 years, do you screen for PA with ARR?			
a) All patients with hypertension	5 (20.0)	3 (5.3)	0.052
b) Patients with hypertension grade 2	2 (8.0)	12 (21.1)	0.208
c) Patients with hypertension grade 3	7 (28.0)	22 (38.6)	0.357
d) Patients with resistant hypertension	18 (72.0)	42 (73.7)	0.862
e) Patients with hypertension and spontaneous hypokalemia	20 (80.0)	49 (86.0)	0.522
f) Patients with hypertension and diuretic-induced hypokalemia	9 (36.0)	13 (22.8)	0.215
g) Patients with hypertension and adrenal incidentaloma	18 (72.0)	35 (61.4)	0.357
h) Patients with hypertension and family history of PA	15 (60.0)	33 (57.9)	0.862
(7) In your daily practice, what are the obstacles to the frequent use of the ARR screening test in a patient with hypertension?			
a) Costs of the exam	2 (8.0)	5 (8.8)	1.000
b) Difficulty in the interpretation of the ARR during interfering medications	12 (48.0)	34 (59.6)	0.327
c) Difficulty to switch an interfering therapy to one without an effect	21 (84.0)	23 (40.4)	**<0.001**
d) Difficulty to perform a confirmatory test in case of a positive ARR	1 (4.0)	15 (26.3)	**0.031**
e) Subtype diagnosis is invasive (AVS) and MRA carries side effects	4 (16.0)	6 (10.5)	0.716
f) Rarity of the disease (futility of the screening in most cases)	2 (8.0)	13 (22.8)	0.133
(8) Which of the following could be useful in increasing the use of the ARR screening test?			
a) None, I perform the test when necessary	9 (36.0)	16 (28.1)	0.471
b) A clinical score which indicates the probability of a patient having this disease	8 (32.0)	21 (36.8)	0.671
c) Having help with the interpretation of ARR results in patients with interfering medication	8 (32.0)	12 (21.1)	0.288
d) It's a rare disease and increasing screening is unnecessary	0 (0.0)	1 (1.8)	1.000
e) The definition of a SOP in our scientific society	7 (28.0)	22 (38.6)	0.357
(10) Which of the following patients do you test for Pheochromocytoma with plasma or urinary fractionated metanephrines?			
a) All patients with hypertension	2 (8.0)	0 (0.0)	0.090
b) Patients with hypertension and tachycardia, sweating and headache	21 (84.0)	41 (71.9)	0.279
c) Patients with hypertensive crises	19 (76.0)	42 (73.7)	0.823
d) Patients with crises induced by steroids, opioids, or beta-blockers	11 (44.0)	21 (36.8)	0.543
e) Patients with resistant hypertension	16 (64.0)	37 (64.9)	0.920
f) Patients with hypertension and adrenal incidentaloma	18 (72.0)	36 (63.2)	0.439
g) All patients with adrenal incidentaloma irrespective of blood pressure levels	10 (40.0)	21 (36.8)	0.791

Responses to questions 6-8 and 10 of the questionnaire after stratification for type of center. Data are reported as absolute numbers and frequencies. A *P*-value less than 0.05 was considered significant and reported in bold characters.

Supplementary Table 4 – Sub-analyses after stratification for geographical area

**Geographical Area**	**North** (n=52)	**Center-South** (n=30)	***P*-value**
(6) Which of the following patients, younger than 65 years, do you screen for PA with ARR?			
a) All patients with hypertension	7 (13.5)	1 (3.3)	0.247
b) Patients with hypertension grade 2	8 (15.4)	6 (20.0)	0.762
c) Patients with hypertension grade 3	17 (32.7)	12 (40.0)	0.632
d) Patients with resistant hypertension	37 (71.2)	23 (76.7)	0.618
e) Patients with hypertension and spontaneous hypokalemia	40 (76.9)	29 (96.7)	**0.026**
f) Patients with hypertension and diuretic-induced hypokalemia	12 (23.1)	10 (33.3)	0.313
g) Patients with hypertension and adrenal incidentaloma	31 (59.6)	22 (73.3)	0.210
h) Patients with hypertension and family history of PA	29 (55.8)	19 (63.3)	0.502
(7) In your daily practice, what are the obstacles to the frequent use of the ARR screening test in a patient with hypertension?			
a) Costs of the exam	4 (7.7)	3 (10.0)	1.000
b) Difficulty in the interpretation of the ARR during interfering medications	29 (55.8)	17 (56.7)	0.920
c) Difficulty to switch an interfering therapy to one without an effect	27 (51.9)	17 (56.7)	0.680
d) Difficulty to perform a confirmatory test in case of a positive ARR	11 (21.2)	5 (16.7)	0.775
e) Subtype diagnosis is invasive (AVS) and MRA carries side effects	6 (11.5)	4 (13.3)	1.000
f) Rarity of the disease (futility of the screening in most cases)	12 (23.1)	3 (10.0)	0.235
(8) Which of the following could be useful in increasing the use of the ARR screening test?			
a) None, I perform the test when necessary	19 (36.5)	6 (20.0)	0.117
b) A clinical score which indicates the probability of a patient having this disease	19 (36.5)	10 (33.3)	0.764
c) Having help with the interpretation of ARR results in patients with interfering medication	9 (17.3)	11 (36.7)	**0.049**
d) It's a rare disease and increasing screening is unnecessary	1 (1.9)	0 (0.0)	1.000
e) The definition of a SOP in our scientific society	16 (30.8)	13 (43.3)	0.252
(10) Which of the following patients do you test for Pheochromocytoma with plasma or urinary fractionated metanephrines?			
a) All patients with hypertension	2 (3.8)	0 (0.0)	0.530
b) Patients with hypertension and tachycardia, sweating and headache	38 (73.1)	24 (80.0)	0.584
c) Patients with hypertensive crises	40 (76.9)	21 (70.0)	0.488
d) Patients with crises induced by steroids, opioids, or beta-blockers	22 (42.3)	10 (33.3)	0.424
e) Patients with resistant hypertension	33 (63.5)	20 (66.7)	0.764
f) Patients with hypertension and adrenal incidentaloma	33 (63.5)	21 (70.0)	0.549
g) All patients with adrenal incidentaloma irrespective of blood pressure levels	21 (40.4)	10 (33.3)	0.527

Answers to questions 6-8 and 10 of the questionnaire after stratification for geographical area. Data are reported as absolute numbers and frequencies. A *P*-value less than 0.05 was considered significant and reported in bold.

Supplementary Table 5 – Sub-analyses after stratification for prevalent specialty

**Prevalent Specialty**	**Internal Medicine** (n=36)	**Cardiology** (n=25)	**Others*** (n=21)	***P*-value**
(6) Which of the following patients, younger than 65 years, do you screen for PA with ARR?				
a) All patients with hypertension	5 (13.9)	2 (8.0)	1 (4.8)	0.639
b) Patients with hypertension grade 2	8 (22.2)	0 (0.0)	6 (28.6)	**0.008**
c) Patients with hypertension grade 3	13 (36.1)	4 (16.0)	12 (57.1)	**0.015**
d) Patients with resistant hypertension	29 (80.6)	18 (72.0)	13 (61.9)	0.304
e) Patients with hypertension and spontaneous hypokalemia	30 (83.3)	21 (84.0)	18 (85.7)	0.851
f) Patients with hypertension and diuretic-induced hypokalemia	13 (36.1)	1 (4.0)	8 (38.1)	**0.004**
g) Patients with hypertension and adrenal incidentaloma	28 (77.8)	10 (40.0)	15 (71.4)	**0.008**
h) Patients with hypertension and family history of PA	22 (61.1)	9 (36.0)	17 (81.0)	**0.009**
(7) In your daily practice, what are the obstacles to the frequent use of the ARR screening test in a patient with hypertension?				
a) Costs of the exam	2 (5.6)	2 (8.0)	3 (14.3)	0.532
b) Difficulty in the interpretation of the ARR during interfering medications	17 (47.2)	15 (60.0)	14 (66.7)	0.323
c) Difficulty to switch an interfering therapy to one without an effect	24 (66.7)	10 (40.0)	10 (47.6)	0.099
d) Difficulty to perform a confirmatory test in case of a positive ARR	10 (27.8)	4 (16.0)	2 (9.5)	0.266
e) Subtype diagnosis is invasive (AVS) and MRA carries side effects	5 (13.9)	1 (4.0)	4 (19.0)	0.289
f) Rarity of the disease (futility of the screening in most cases)	3 (8.3)	10 (40.0)	2 (9.5)	**0.006**
(8) Which of the following could be useful in increasing the use of the ARR screening test?				
a) None, I perform the test when necessary	14 (38.9)	5 (20.0)	6 (28.6)	0.283
b) A clinical score which indicates the probability of a patient having this disease	11 (30.6)	12 (48.0)	6 (28.6)	0.282
c) Having help with the interpretation of ARR results in patients with interfering medication	9 (25.0)	5 (20.0)	6 (28.6)	0.807
d) It's a rare disease and increasing screening is unnecessary	0 (0.0)	1 (4.0)	0 (0.0)	0.561
e) The definition of a SOP in our scientific society	11 (30.6)	10 (40.0)	8 (38.1)	0.715
(10) Which of the following patients do you test for Pheochromocytoma with plasma or urinary fractionated metanephrines?				
a) All patients with hypertension	1 (2.8)	1 (4.0)	0 (0.0)	1.000
b) Patients with hypertension and tachycardia, sweating and headache	31 (86.1)	14 (56.0)	17 (81.0)	**0.025**
c) Patients with hypertensive crises	27 (75.0)	19 (76.0)	15 (71.4)	0.932
d) Patients with crises induced by steroids, opioids, or beta-blockers	17 (47.2)	4 (16.0)	11 (52.4)	**0.014**
e) Patients with resistant hypertension	25 (69.4)	10 (40.0)	18 (85.7)	**0.004**
f) Patients with hypertension and adrenal incidentaloma	26 (72.2)	10 (40.0)	18 (85.7)	**0.003**
g) All patients with adrenal incidentaloma irrespective of blood pressure levels	18 (50.0)	4 (16.0)	9 (42.9)	**0.018**

Responses to questions 6-8 and 10 of the questionnaire after stratification according to prevalent specialty. Data are reported as absolute numbers and frequencies. A *P*-value less than 0.05 was considered significant and reported in bold. *Others refers to: endocrinology, nephrology and geriatrics.

Supplementary Table 6 – Stratification for prevalent specialty (sub-analysis on Endocrinology)

Prevalent Specialty	Internal Medicine (n=36)	Cardiology (n=25)	Endocrinology (n=8)	Others* (n=13)	*P*-value
(6) Which of the following patients, younger than 65 years, do you screen for PA with ARR?					
a) All patients with hypertension	5 (13.9)	2 (8.0)	1 (12.5)	0 (0.0)	0.581
b) Patients with hypertension grade 2	8 (22.2)	0 (0.0)	3 (37.5)	3 (23.1)	**0.011**
c) Patients with hypertension grade 3	13 (36.1)	4 (16.0)	7 (87.5)	5 (38.5)	**0.003**
d) Patients with resistant hypertension	29 (80.6)	18 (72.0)	7 (87.5)	6 (46.2)	0.100
e) Patients with hypertension and spontaneous hypokalemia	30 (83.3)	21 (84.0)	6 (75.0)	12 (92.3)	0.769
f) Patients with hypertension and diuretic-induced hypokalemia	13 (36.1)	1 (4.0)	4 (50.0)	4 (30.8)	**0.005**
g) Patients with hypertension and adrenal incidentaloma	28 (77.8)	10 (40.0)	7 (87.5)	8 (61.5)	**0.011**
h) Patients with hypertension and family history of PA	22 (61.1)	9 (36.0)	7 (87.5)	10 (76.9)	**0.021**
(7) In your daily practice, what are the obstacles to the frequent use of the ARR screening test in a patient with hypertension?					
a) Costs of the exam	2 (5.6)	2 (8.0)	1 (12.5)	2 (15.4)	0.561
b) Difficulty in the interpretation of the ARR during interfering medications	17 (47.2)	15 (60.0)	5 (62.5)	9 (69.2)	0.521
c) Difficulty to switch an interfering therapy to one without an effect	24 (66.7)	10 (40.0)	3 (37.5)	7 (53.8)	0.157
d) Difficulty to perform a confirmatory test in case of a positive ARR	10 (27.8)	4 (16.0)	1 (12.5)	1 (7.7)	0.440
e) Subtype diagnosis is invasive (AVS) and MRA carries side effects	5 (13.9)	1 (4.0)	3 (37.5)	1 (7.7)	0.096
f) Rarity of the disease (futility of the screening in most cases)	3 (8.3)	10 (40.0)	0 (0.0)	2 (15.4)	**0.010**
(8) Which of the following could be useful in increasing the use of the ARR screening test?					
a) None, I perform the test when necessary	14 (38.9)	5 (20.0)	3 (37.5)	3 (23.1)	0.390
b) A clinical score which indicates the probability of a patient having this disease	11 (30.6)	12 (48.0)	2 (25.0)	4 (30.8)	0.510
c) Having help with the interpretation of ARR results in patients with interfering medication	9 (25.0)	5 (20.0)	1 (12.5)	5 (38.5)	0.555
d) It's a rare disease and increasing screening is unnecessary	0 (0.0)	1 (4.0)	0 (0.0)	0 (0.0)	0.561
e) The definition of a SOP in our scientific society	11 (30.6)	10 (40.0)	5 (62.5)	3 (23.1)	0.267
(10) Which of the following patients do you test for Pheochromocytoma with plasma or urinary fractionated metanephrines?					
a) All patients with hypertension	1 (2.8)	1 (4.0)	0 (0.0)	0 (0.0)	1.000
b) Patients with hypertension and tachycardia, sweating and headache	31 (86.1)	14 (56.0)	7 (87.5)	10 (76.9)	0.053
c) Patients with hypertensive crises	27 (75.0)	19 (76.0)	8 (100.0)	7 (53.8)	0.137
d) Patients with crises induced by steroids, opioids, or beta-blockers	17 (47.2)	4 (16.0)	5 (62.5)	6 (46.2)	**0.026**
e) Patients with resistant hypertension	25 (69.4)	10 (40.0)	7 (87.5)	11 (84.6)	**0.014**
f) Patients with hypertension and adrenal incidentaloma	26 (72.2)	10 (40.0)	7 (87.5)	11 (84.6)	**0.010**
g) All patients with adrenal incidentaloma irrespective of blood pressure levels	18 (50.0)	4 (16.0)	6 (75.0)	3 (23.1)	**0.004**

Answers to questions 6-8 and 10 after stratification for specialty, including Internal Medicine, Cardiology, Endocrinology and others. Data are reported as absolute number (frequency). A *P*-value less than 0.05 was considered significant and reported in bold. *Others refers to nephrology and geriatrics.
